# Unintended consequences of technology in competency-based education: a qualitative study of lessons learned in an OtoHNS program

**DOI:** 10.1186/s40463-023-00649-2

**Published:** 2023-08-23

**Authors:** Mary Ott, Tavis Apramian, Sayra Cristancho, Kathryn Roth

**Affiliations:** 1 Centre for Education Research and Innovation, Schulich School of Medicine and Dentistry, Western University, London, Canada; 2 Division of Palliative Care, Department of Family & Community Medicine, University of Toronto, Toronto, Canada; 3 Department of Otolaryngology-Head and Neck Surgery, Schulich School of Medicine and Dentistry, Western University, London, Canada

**Keywords:** Assessment, CBME, e-portfolio, Technology design, Unintended consequences

## Abstract

**Background:**

Formative feedback and entrustment ratings on assessments of entrustable professional activities (EPAs) are intended to support learner self-regulation and inform entrustment decisions in competency-based medical education. Technology platforms have been developed to facilitate these goals, but little is known about their effects on these new assessment practices. This study investigates how users interacted with an e-portfolio in an OtoHNS surgery program transitioning to a Canadian approach to competency-based assessment, Competence by Design.

**Methods:**

We employed a sociomaterial perspective on technology and grounded theory methods of iterative data collection and analysis to study this OtoHNS program’s use of an e-portfolio for assessment purposes. All residents (n = 14) and competency committee members (n = 7) participated in the study; data included feedback in resident portfolios, observation of use of the e-portfolio in a competency committee meeting, and a focus group with residents to explore how they used the e-portfolio and visualize interfaces that would better meet their needs.

**Results:**

Use of the e-portfolio to document, access, and interpret assessment data was problematic for both residents and faculty, but the residents faced more challenges. While faculty were slowed in making entrustment decisions, formative assessments were not actionable for residents. Workarounds to these barriers resulted in a “numbers game” residents played to acquire EPAs. Themes prioritized needs for searchable, contextual, visual, and mobile aspects of technology design to support use of assessment data for resident learning.

**Conclusion:**

Best practices of technology design begin by understanding user needs. Insights from this study support recommendations for improved technology design centred on learner needs to provide OtoHNS residents a more formative experience of competency-based training.

**Graphical abstract:**

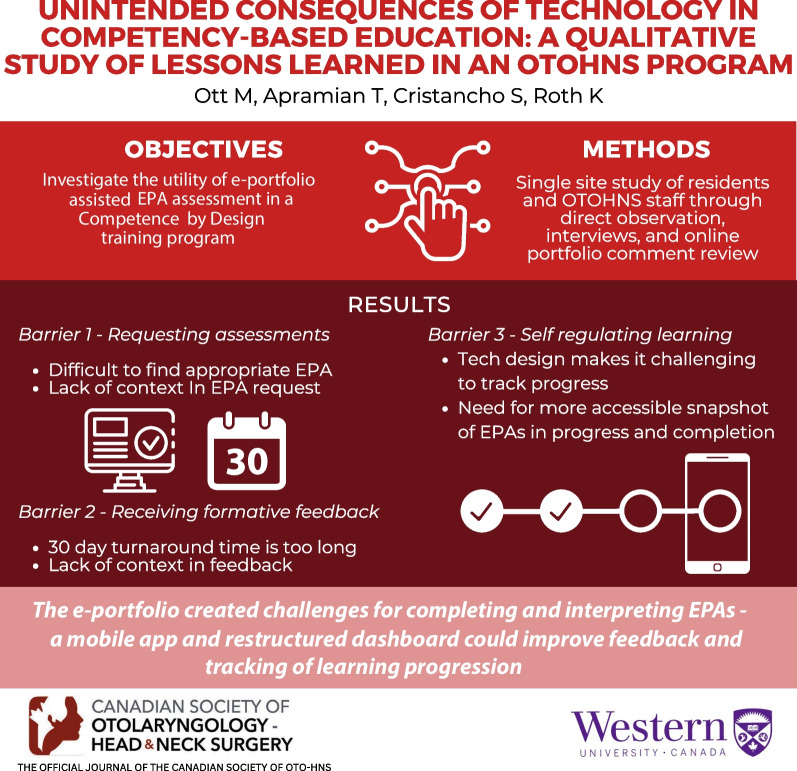

## Introduction

Programmatic assessment in competency-based medical education (CBME) is a multi-faceted approach intended to support the development of competency [1–3]. When learners know what they need to do to improve, they can begin to self-regulate their learning [4–6]. Formative feedback from frequent assessments of entrustable professional activities (EPAs) should provide learners this growth-oriented information [3–6]. Entrustment ratings on EPAs also contribute numerous data points to assist competency committees in making summative decisions about learner progression [2, 3, 7–9]. However, due to the ongoing nature of EPA assessment in clinical learning settings, there is a burgeoning amount of data to document and analyze. Advocates for CBME have urged the development of e-portfolios to facilitate the aims of programmatic assessment [10–13]. Are these technologies living up to their promise?

Complex educational reforms require nuanced attention to core values to implement with fidelity in diverse contexts [1, 2, 14, 15]. There is a strong focus in the CBME literature on ways to mitigate human factors that may thwart intended outcomes. Discussions after implementation have shown the need for improvement in change management [1, 12, 14, 15], faculty development [2, 16, 17], and orientation of learners [18, 19]. When it comes to technology, solutions are presented for the human challenges of managing the data generated by EPA assessments [20–23]. However, little research has explored the role technology may play in creating unintended consequences for CBME [24, 25]. This leaves a blind spot for implementation, because technology often has unpredictable or unintended effects in practice [26–28].

In Canada, Otolaryngology-Head & Neck (OtoHNS) residency programs were among the first to implement Competence by Design (CBD), an approach to competency-based postgraduate medical education mandated by the Royal College of Physicians and Surgeons of Canada. As early adopters, OtoHNS programs were required to use an e-portfolio developed by the Royal College. Since implementation in 2017, a longitudinal survey of learner experience with CBD has identified unintended effects on residents, including difficulties that residents in OtoHNS programs and others have encountered with use of this e-portfolio [29].

Research that investigates the effects of technology in practice may contribute insight to this problem. For example, Suchman, a pioneer in the field of human-technology interaction, drew on ethnographic methods and sociomaterial perspectives to show that unproductive workarounds are the result of communication barriers between users and technology [27]. A sociomaterial lens draws attention to the agency of material ‘actors’ to position us in practice, acting with and on us [30–32]. Following Suchman’s example [27, 28], this study sought an in-depth understanding of the effects of the e-portfolio on assessment practices in a Canadian OtoHNS program.

## Methods

This study employed situational analysis, a sociomaterial approach to grounded theory using multiple methods of data collection such as document review, observation, interviews, and visualizations to understand the effects of actors in a practice [33, 34]. Grounded theory is suited to the study of educational situations because it generates understandings of how practice emerges through social processes. A sociomaterial approach to research considers material, such as technology, as an *actor* in social processes because material has agency in structuring how humans interact with it and with one another. Like other grounded theory methodologies, situational analysis takes an iterative approach to data collection and analysis, seeking conceptual depth by theoretically sampling new data for developing insights [35–37]. Themes and relationships in the data are identified to develop an explanatory concept of how human and material actors interact to shape practice in the situation under study [33, 34].

### Study context

Since OtoHNS was among the first specialties to implement CBD, we chose a focal program to study new assessment practices to afford a rich picture of implications for faculty and learners. The OtoHNS program at Western University is mid-sized by Canadian standards, functions as its own academic department, and is a sought-after training program that attracts high calibre residents. The size, organizational and educational conditions of this program made it an ideal ‘critical case sample’ [38] for an in-depth study of a ‘best-case’ scenario.

### Participants and data sources

All residents (n = 14) and competency committee members (n = 7) in the program consented to participate in the study. Four residents consented to analysis of anonymized feedback from their e-portfolios. Faculty consented to collection of anonymized observational fieldnotes. Verbatim quotes from members of the competency committee are indicated by S1, S2 in the results. The audio-recorded focus group with residents occurred during an academic day, with 11 residents present. Due to some overlap in data sources, the total number of residents in the study was 14. The focus group transcript was anonymized, with residents indicated by numbered references in the results (R1, R2, and so on).

### Data collection and analysis

Analysis included the content of EPA feedback in the e-portfolios, the discussion of this data in the competency committee meeting, and a resident focus group. Questions for the focus group concerned use of feedback to self-regulate learning, such as how residents decided who to ask and when to ask for EPA assessments, how they used the feedback to gauge their progression, and how they used the e-portfolio to facilitate this process. Since the observation of the competency committee raised faculty concerns with the design of the e-portfolio, the process of theoretical sampling compelled us to further explore the agency of this technology in the documentation and use of EPA data. As part of the focus group, we asked the residents to draw pictures of e-portfolio interfaces that could improve the experience of using EPA data for learning. Conceptual depth was achieved through ongoing analytic meetings with other members of the research team to consider the adequacy of the data to support this theory. We checked for resonance of the findings [38] with the faculty and residents in the program through presentations at department research days both during and after the study concluded. The figures of e-portfolio screens in the results are an encapsulation of the visual and textual data in the focus group and competency committee observation, prepared with the assistance of a graphic artist.

## Results

The e-portfolio created barriers to the assessment practices of documenting, accessing, and interpreting EPA data for both faculty and residents, but the residents faced more challenges. Difficulties collating and visualizing EPA data slowed the capacity of competency committee members to review resident performance in depth. However, residents faced three obstacles to use of the e-portfolio: requesting assessments, receiving formative feedback, and using data to self-regulate learning. The workload of trying to manage EPA data led to unintended workarounds for these barriers, resulting in a “numbers game” (R7) residents played to acquire successful EPA assessments.

**Fig. 1:**
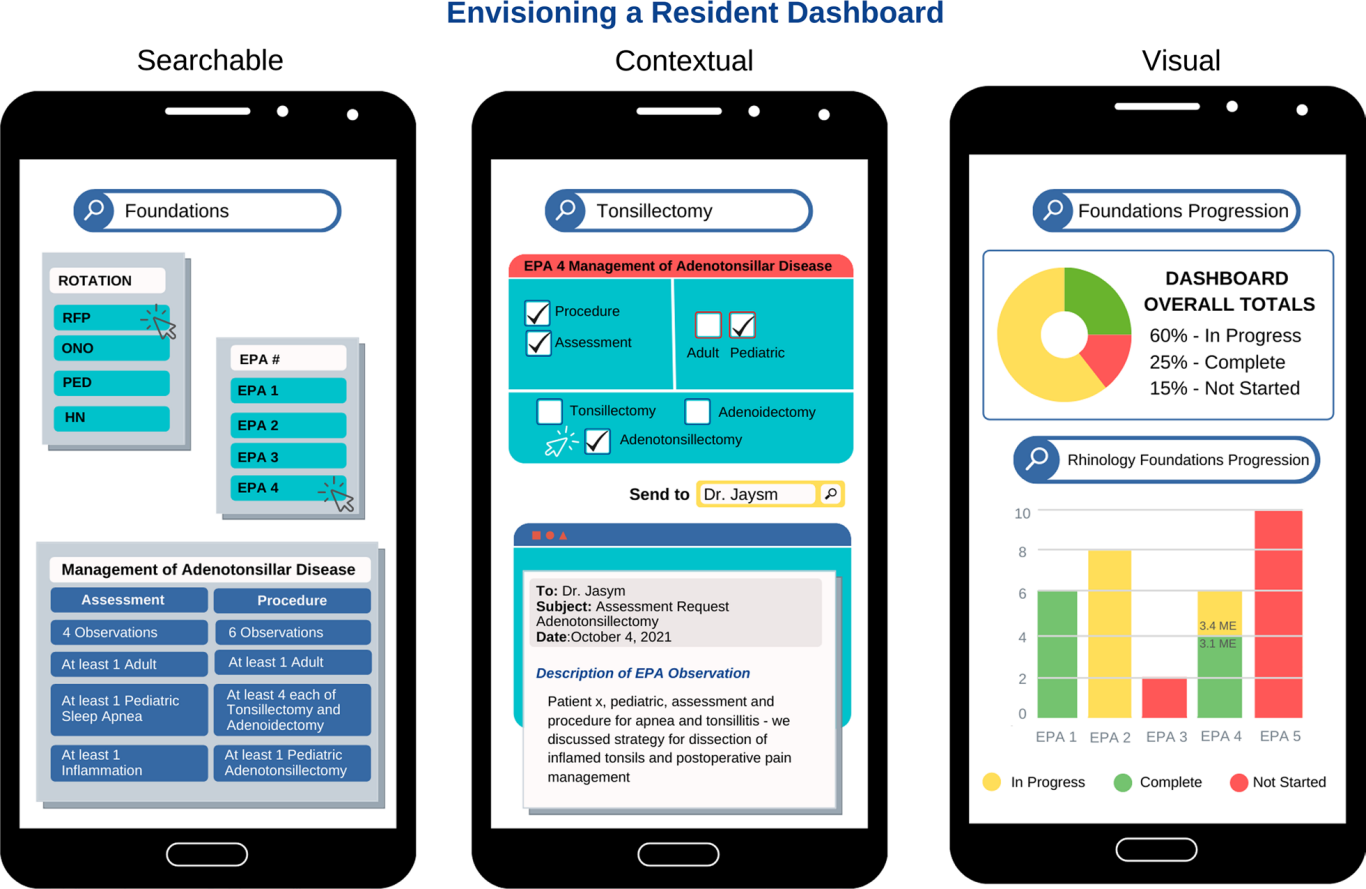
Envisioning a resident dashboard

The findings are organized to detail each technology barrier and resulting workarounds. Figure 1 illustrates themes in the data that prioritized needs for searchable, contextual, visual, and mobile technology solutions to overcome these challenges.

### Requesting EPA assessments

Residents described difficulties requesting EPAs through the e-portfolio that mapped onto the procedures they were learning. One commented, “the stem of the problem is, the website is not friendly, EPAs are not designed properly” (R8). Another explained, “you have to find which is the appropriate EPA, which is not always very obvious. For example, this one about hearing loss is actually the tube insertion one” (R2). The design visualizations of improved interfaces showed that how an EPA is structured in the curriculum plan matters for how residents search for it in the e-portfolio. Their sketches illustrated that organizing EPAs in the database in the same way they are laid out in their program curriculum map, by level of training and rotation, would helpfully narrow the search field to “the appropriate EPA”. The searchable schematic in Fig. 1 encapsulates these ideas.

Residents also felt hampered by the extra work required to manage EPA feedback requests and notifications within the e-portfolio: “You hit request, and then it generates a note to the staff who then sees, ‘Request for EPA Number 3.7’… they don’t get any other information than that” (R2). To prompt faculty to recall the case for feedback, the residents developed communicative workarounds: “you have to either communicate that to them in person and they have to remember, or you have to send a separate email telling them, hey, I’m sending you an EPA, it’s about patient X, Y, Z” (R2). Residents agreed that this tactic of sending extra emails and reminders was essential to ensure that faculty understood which procedure they were being asked to provide feedback on and to complete assessment documentation. However, while residents had to shoulder the workload of filling in contextual gaps for faculty, they faced the same problem of lack of context when receiving feedback from the system.

### Receiving formative feedback

The residents described the feedback notifications as “generic”, making it difficult to remember which cases they could be related to.

It’s just a very generic email so, it doesn’t say what you’re being evaluated, it just says so-and-so completed an evaluation on your form. You have to think, 30 days later, you have to think about what did you send them? (R7)

The default setting of up to “30 days” from sending a request to receiving feedback was intended to allow faculty time to complete assessments. However, both the delay of information and lack of context rendered the feedback uninformative, as the following conversation between residents highlights:

R4: I, over time, I just stopped reading the EPA feedback… I mean, just delete it from my inbox. I guess, yeah, it doesn’t tell me the exact contextual context. I know that I achieved it when I asked so I don’t read the feedback for it.

There were two reasons residents found feedback documented in the e-portfolio did not support their learning. First, as Resident 7 pointed out, “30 days later” the assessment served a purely summative, “black or white”, purpose. Residents found the feedback lacked specificity on ways to improve performance. Our own analysis of EPA feedback in the residents’ e-portfolios confirmed that details were scant: “good economy of motion”, “good understanding of relevant anatomy”, “knew all components of the procedure”.

But there was another reason feedback was uninformative. Resident 4’s commentary on why emails with feedback notifications were deleted is telling: “I know that I achieved it when I asked for it”. The residents described a process of waiting until they were reasonably assured they would receive a ‘successful’ entrustment rating before sending feedback requests for an EPA: “When?—confident on procedure, never first-time doing procedure” (R1).

It might seem that residents were hesitant to receive more improvement-focussed assessment. However, when asked how residents decided who to ask and when to ask for EPA assessments, the answer landed on a tactic for efficiency rather than avoidance of constructive feedback:

It’s basically a numbers game. You’re like, are you going to send one out that’s got 30 days and then you’re going to have to re-request it? Probably not. It’s not a good use of your time, it’s not a good use of their time. (R7)

Given the high stakes of the “numbers game” for progression, it might also seem that residents would seek feedback strategically from faculty known to give higher entrustment ratings. However, as the following exchange outlines, this was not the case.

R11: I would say, I just ask based on people who I know will get it back to me and people who are willing to do it.

R2: And most staff will really only happily do one EPA for you a day.

To protect their time and faculty time in the workload of CBD, residents managed the numbers game by deciding who to ask based on faculty approachability and efficiency and deciding when to ask once reasonably confident of success. The contextual features in the second schematic in Fig. 1 show how the workload of managing the numbers game could be reduced and the value of feedback increased with a technology design that included contextual details in feedback requests.

### Using assessment data to self-regulate learning

Faculty and residents also shared that the technology design made it difficult to track progression towards entrustment on EPAs. In the CBD curriculum, EPAs have a number of components; for example, entrustment of the tonsillectomy EPA required a variety of patient indications and multiple assessment observations. In the competency committee meeting, members had to toggle between different screens to see how many EPA observations were complete, which contextual variables were incomplete, and to read the feedback on the observations. Faculty struggled to interpret this data holistically. The following exchange between two committee members indicates that faculty understood accessibility of EPA data was a problem for the residents as well:

S2: They need a system for logging themselves—they can’t see them, we struggle because we’re flipping back and forth between screens.

S6: If they could just have a personal dashboard so they know! It’s so hard to keep track.

The problem residents faced in tracking their progression was piecing contextual variables together, finding opportunities on different days to complete EPA requirements. The challenge to “keep track” was compounded by the absence of interpretive details in the reporting system.

As Resident 11 explained, notifications of a “pass” for an EPA assessment did not provide information on which contextual variables “contributed to your pass”. The schematic for visual features in Fig. 1 illustrates the residents’ requests for a more accessible snapshot of EPAs in progress and more informative metrics on completion of contextual variables.

## Discussion

Barriers posed by the e-portfolio to requesting EPA assessments, receiving actionable feedback, and self-regulating learning had unintended consequences for the residents in this study. The formative intentions of CBME did not translate to practice, and the work of achieving EPAs was reduced to a numbers game for acquiring successful observations before assessment requests timed out in the system. While the burden of workarounds to the design of the interface fell mainly on the residents, their experiences struggling to access and interpret EPA data were shared by the competence committee. This study resonates with research on workload for a competence committee at a different institution [24]. While this committee used a different technology interface, similar issues with visualization of data slowed capacity to review trainee performance. The Royal College’s own program evaluation of CBD confirms these findings, taking note of calls to improve the design of e-portfolios [39]. However, to avoid the quick fixes of ‘solutionism’ [26, 40] it is essential to understand that the unintended consequences of technology in this case are more than a problem of workload. Insights from our study support recommendations for enhanced design of technology to improve the quality and accessibility of EPA data for learning.

For example, lack of context in EPA requests and notifications posed the largest obstacle to assessment workflow for residents. But the time delays created and communicative workarounds to fill in contextual gaps led to the emergence of efficiency strategies for acquisition of EPA assessments that had little to do with seeking growth-oriented feedback. While improved search functions, contextual details, and push notifications would ease the workload of CBD, we suggest that mobile designs for EPA assessment could go furthest to address the problem of formative assessment. A possible productive workaround using the current version e-portfolio is to have residents pre-fill EPA assessments to the point that faculty feedback can be documented in the moment. However, in an example of truly mobile design for CBME, researchers found that an assessment app increased context specificity and engagement with feedback significantly [21]. A pilot study of a mobile assessment app in an OtoHNS program likewise demonstrates the feasibility of this approach for operative settings [41].

We also showed that lack of clarity in how EPA data was displayed in the e-portfolio limited the ability of residents to monitor their progression. The problem of human–machine communication is well known [27]. Visual dashboards have been developed as a sign language of sorts to facilitate this interaction [42, 43], and user design has emerged as an important field relying on qualitative research methods to empathize with user needs and optimize solutions [20–23, 44]. In a noteworthy example in the CBD context, researchers co-designed a visual dashboard with residents to support self-regulated learning through improved functions to access and interpret their EPA data [23].

Since best practices of user design begin by understanding user needs, this raises a critical issue. The needs of competence committees making entrustment decisions are different from the needs of learners for assessment data that can support their learning. We see this in research focused on meeting competence committee needs [10, 20, 24], and in our own experience. Due to the challenges with the Royal College’s e-portfolio, our institution is developing a different technology solution and has prioritized design of a visual dashboard for competence committees. Designing technology to meet competence committee needs first may have the unintended consequence of raising the stakes of summative assessment over formative assessment. This priority may communicate to residents that what matters most is efficient acquisition of ‘successful’ EPAs. If we value the importance of learner-centred medical education [1, 4, 6] and the role that formative feedback plays in this process [3–6], then we must design solutions for CBME that prioritize assessment for learners. This resonates with the recent Royal College Resident Pulse Check 2022 [19], demonstrating issues with electronic portfolios and workplace-based EPA assessment. The impact of EPA acquisition on resident wellness is an immediate priority arising from this document and our observations highlight some feasible solutions.

### Limitations

This research employs a purposefully small sample of a particular context for competency-based medical education. While this allows for in-depth analysis, transferability is limited to programs with similar contexts. Additionally, focus groups may converge on similar experiences, which can exclude disconfirming data. Research using individual interviews could provide further confirmation.

## Conclusion

Competency-based medical education has the potential to improve residency training through improved feedback and entrustment practices, and technology plays a key role in managing assessment data to support these goals of programmatic assessment. However, this study of an OtoHNS program transitioning to a competency-based curriculum demonstrates that technology design may obstruct these purposes unintentionally by making EPA data difficult for faculty and residents to document, access, and interpret. We also showed that the challenges for residents can have a weightier impact, increasing their workload and making it more difficult to self-regulate their learning. This study provides insight into how technology design centered on learner needs could provide residents a more formative experience of competency-based training.

## Data Availability

Due to the identifiable nature of handwriting in the visual data and setting details included in the unredacted focus group transcript, we have not made these data publicly available. Instead, we have opted to use representative images and anonymized quotes in the results.
